# Chrysotile Asbestos and Mesothelioma

**DOI:** 10.1289/ehp.1002446

**Published:** 2010-07

**Authors:** Richard A. Lemen

**Affiliations:** U.S. Public Health Service (retired), National Institute for Occupational Safety Health (retired), Canton, Georgia, E-mail: richard@ralemen.org

The Editor’s Summary for the article by Tse et al. (2010) stated the following:

Assuming an average latency of 42 years, the authors predict that incidence rates will peak in 2009 and that diagnoses will peak in 2014. However, they caution that ongoing use of chrysotile asbestos (which has been implicated but not conclusively established as a cause of mesothelioma) and the release of asbestos fibers from older buildings during demolition or renovation may slow the projected decline.

The statement concerning chrysotile asbestos being “implicated but not conclusively established as a cause of mesothelioma” is inconsistent with current scientific opinion. I refer you to the most recent evaluation by the International Agency for Research on Cancer in which Straif et al. (2009) stated,

Epidemiological evidence has increasingly shown an association of all forms of asbestos (chrysotile, crocidolite, amosite, tremolite, actinolite, and anthophyllite) with an increased risk of lung cancer and mesothelioma. Although the potency differences with respect to lung cancer or mesothelioma for fibres of various types and dimensions are debated, the fundamental conclusion is that all forms of asbestos are “carcinogenic to humans” (Group 1).

In addition, opinions such as that expressed in the Editor’s Summary are advanced only by scientists with prochrysotile industry bias.

When I wrote the draft for the first *IARC Monograph* on asbestos in 1976, which the expert committee accepted and published in 1977 as *IARC Monograph* Volume 14, a similar conclusion was stated: “Many pleural and peritoneal mesotheliomas have been observed after occupational exposure to crocidolite, amosite and chrysotile.” Since then—more than 30 years—science has not changed its opinion that all forms of asbestos, including chrysotile, cause mesothelioma.

In fact, in the article that is the subject of the Editor’s Summary, Tse et al. (2010) did not indicate that chrysotile is not a cause of mesothelioma; on the contrary, they stated the following:

Although the mesothelioma incidence is anticipated to decline in the coming decades, it may not decrease to background risk levels given that chrysotile consumption has not been banned under the current legislation and that secondary asbestos exposure from the environment will likely continue. Nevertheless, the hypotheses generated from this ecologic study need further confirmation by subsequent analytic studies. The present study provides supportive evidence for an immediate and global ban on asbestos use.

I hope that future Editor’s Summaries will reflect the conclusions of the article and not put forth statements that are not supported by mainstream science. I also support the conclusion of Tse e al. (2010) for “an immediate and global ban on asbestos use.”
